# Seeking new approach for therapeutic treatment of cholera disease via inhibition of bacterial carbonic anhydrases: experimental and theoretical studies for sixteen benzenesulfonamide derivatives

**DOI:** 10.1080/14756366.2019.1618292

**Published:** 2019-07-08

**Authors:** Rosaria Gitto, Laura De Luca, Francesca Mancuso, Sonia Del Prete, Daniela Vullo, Claudiu T. Supuran, Clemente Capasso

**Affiliations:** a Department of Chemical, Biological, Pharmaceutical and Environmental Sciences (CHIBIOFARAM), University of Messina, Messina, Italy;; b Department of Biology, Agriculture and Food Sciences, Institute of Biosciences and Bioresources- CNR, Napoli, Italy;; c NUROFARBA Department, University of Florence, Sesto Fiorentino, Italy

**Keywords:** Carbonic anhydrase inhibitors (CAIs), benzenesulfonamides, *Vibrio cholera*, molecular docking

## Abstract

A series of sixteen benzenesulfonamide derivatives has been synthesised and tested as inhibitors of *Vibrio cholerae* carbonic anhydrase (CA) enzymes, belonging to *α*-CA, *β*-CA, and *γ*-CA classes (VchCA*α*, VchCA*β*, and VchCA*γ*). The determined *K*_i_ values were compared to those of selected human CA isoforms (hCA I and hCA II). Structure-affinity relationship analysis highlighted that all tested compounds proved to be active inhibitors of VchCA*α* at nanomolar concentration. The VchCA*β* activity was lower to respect inhibitory efficacy toward VchCA*α*, whereas, these benzenesulfonamide derivatives failed to inhibit VchCA*γ*. Interestingly, compound **7e** combined the best activity toward VchCA*α* and VchCA*β*. In order to obtain a model for binding mode of our inhibitors toward bacterial CAs, we carried out docking simulations by using the available crystal structures of VchCA*β*.

## GRAPHICAL ABSTRACT

**Figure:**
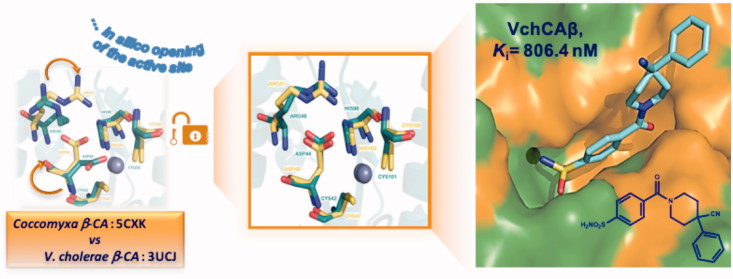

## 1. Introduction

The gram-negative bacterium *Vibrio cholerae* is responsible for the secretory diarrheal disease cholera that infects millions of people worldwide and might cause high mortality if left untreated. While the rehydration therapy is an effective treatment approach as well as the use of antibiotics in severe infections, there is a pressing need for agents that prophylactically could prevent the injurious effects of a cholera infection.

**Figure 1.**
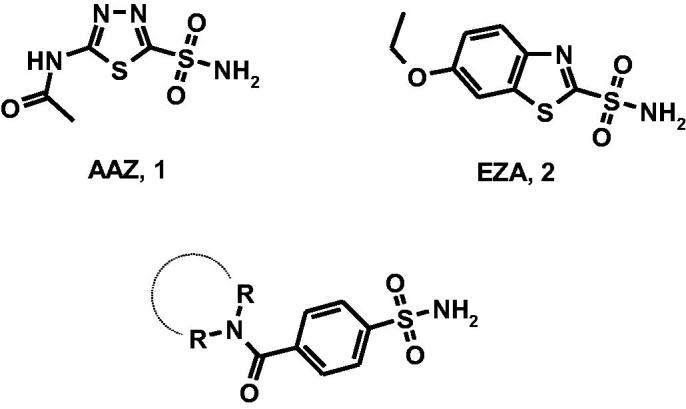
Chemical structures of well-known CAIs acetazolamide (AAZ, **1**) and ethoxzolamide (EZA, **2**) and designed 4-(cycloalkyl)-1-carbonylbenzenesulfonamides.

There is evidence that protein ToxT is one of the transcription factors activating the *V. cholerae* virulence gene expression; therefore, ToxT is a target for the development of cholera therapeutics^1^^,^^2^. ToxT activity is (a) negatively controlled by the unsaturated fatty acids (UFAs) of the bile and (b) enhanced by the presence of bicarbonate in the upper small intestine where *V. cholerae* preferentially colonises. Therefore, bicarbonate is likely an important chemical signal during the cholera disease^3^^,^^4^. Generally, bacteria increase cytosolic bicarbonate levels through bicarbonate transporter proteins and carbonic anhydrases (CAs, EC 4.2.1.)^2^. It has been demonstrated that *V. cholerae* can increase the cytosolic bicarbonate levels through the hydration of CO_2_ by the action of CAs since *V. cholerae* lacks bicarbonate transporter proteins in its genome^3^. Thus, the CAs represent an attractive molecular target for the development of innovative anti-infective agents^3^. The metalloenzyme CAs are grouped into seven genetically distinct families, named α-, β-, γ-, δ-, ζ-, η-, and ɵ-CAs, with different structures and active site architecture as well as metal ions in the catalytic region (zinc, iron, cobalt, and cadmium)^5^. *Vibrio cholerae* encodes carbonic anhydrase belonging to α-CA, β-CA, and γ-CA classes (VchCAα, VchCAβ, and VchCAγ)^6^, that could represent potential targets for the development of anti-infectives targeting *V. cholerae* colonisation^7–9^. The α- and β-CAs are Zn(II) metalloenzymes, whereas γ-CAs use Fe(II) as metal ion; however they are also active with Zn(II) or Co(II). The metal ion is coordinated by three His residues in the α- and γ-classes, and one His and two Cys residues in the β-class. Several classes of CA inhibitors (CAIs)^10^^,^^11^ have been identified; among them, sulfonamide derivatives have been shown to anchor the metal-coordinated water molecule/hydroxide ion within catalytic site thus inducing CA inhibition. The prototype sulfonamide inhibitor acetazolamide (Figure 1, AAZ, **1**) is clinically used as diuretic and anticonvulsant agent in humans; additionally, AAZ displays inhibitory effects toward bacterial isozymes. Moreover, as confirmation of the role of bicarbonate in virulence and colonisation processes, the carbonic anhydrase inhibitor ethoxzolamide (EZA, **2**) proved to decrease the virulence gene expression and to reduce the growth of pathogen^7^.

The principal drawback using CAIs as antiinfectives agents is the lack of selectivity towards the pathogenic versus human isoforms^7^. For this reason, many research groups are continually involved in the synthesis of new CAIs or in the modification/optimisation of the existing inhibitors, which are commonly tested on CAs from mammalian and pathogenic organisms^10^^,^^11^. Most of these inhibitors represent interesting leads towards the optimisation of new antibiotic agents showing excellent inhibitory efficiency and selectivity for the target CAs over the human (h) off-target isoform hCA I^10^^,^^11^. In the course of our efforts to identify novel selective CAIs, we have synthesised and tested a series of quinolone/isoquinoline-arylsulfonamides showing high affinity toward human CAs^12–22^. Specifically, we have reported the discovery of a set of heteroaryl-N-carbonylbenzenesulfonamides as a class of potent inhibitors of druggable CA isoforms (hCA II, hCA VII, hCA IX, and hCA XIV)^15^^,^^17^^,^^18^^,^^20–22^.

Recently, we have studied a new small library of CAIs containing azepine/piperidine/piperazine nucleus linked to benzenesulfonamide fragment and disclosed several compounds that demonstrated inhibitory effects in low nanomolar range toward hCAs^23^. This series of compounds has been rationally designed by using the cycloalkylamine nucleus as the core for binding contact in the middle area of catalytic site (Figure 1). The visual inspection of their binding mode in co-crystal adducts with hCA II and hCA VII confirmed that the sulfonamide portion is crucial for CA recognition process^23^. By considering the high inhibitory effects of this series of potent sulfonamide compounds, we decided to further study their CA inhibitory profile thus exploiting their capability to act as potential anti-infective agents. Therefore, the primary purpose of our work is to provide new information about structure-affinity relationship relative to the inhibition of the three classes from *V. cholerae,* VchCAα, VchCAβ, and VchCAγ . Particularly, our idea was to study in depth the role of hydrophobic interactions within VchCA cavity.

## 2. Materials and methods

### 2.1. Chemistry

All reagents were used without further purification and bought from common commercial suppliers. Microwave-assisted reactions were carried out in a Focussed Microwave TM Synthesis System, Model Discover (CEM Technology Ltd Buckingham, UK). Melting points were determined on a Buchi B-545 apparatus (BUCHI Labortechnik AG Flawil, Switzerland) and are uncorrected. By combustion analysis (C, H, N) carried out on a Carlo Erba Model 1106-Elemental Analyser we determined the purity of synthesised compounds; the results confirmed a ≥ 95% purity. Merck Silica Gel 60 F254 plates were used for analytical TLC (Merck KGaA, Darmstadt, Germany). For detection, iodine vapour and UV light (254 nm) were used. Flash Chromatography (FC) was carried out on a Biotage SP1 EXP (Biotage AB Uppsala, Sweden). ^1^H NMR spectra were measured in dimethylsulfoxide-d6 (DMSO-d_6_) and CDCl_3_ with a Varian Gemini 300 spectrometer (Varian Inc. Palo Alto, California, USA); chemical shifts are expressed in δ (ppm) and coupling constants (*J*) in hertz. All exchangeable protons were confirmed by addition of D_2_O. R_f_ values were determined on TLC plates using a mixture of DCM/MeOH (96/4) as eluent. All compounds have been re-synthesised following the previously reported procedure, that is briefly described below^23^.

#### 2.1.1. Synthetic procedures for benzenesulfonamides 5a–e, 6a, 7a–f, 8a–d

**Pathway i:** To a solution of 4-(aminosulfonyl)benzoic acid (**4**) (6 mmol) in THF (15 ml) the carbonylimidazole (6 mmol) (CDI) at 0 °C was added. The obtained mixture was stirred at room temperature for 3 h and then the appropriate amine derivative (15 mmol) in DMF (5 ml) was added dropwise. The reaction mixture was stirred at room temperature for 2 h. The solvent was removed *in vacuo*; by adding of aqueous solution of NaHCO_3_ (5 ml) we obtained compounds **5a** and **5d** as crude products, which were purified through crystallization by a mixture of Et_2_O and EtOH (1:1)

**Pathway ii:** A mixture of 4-(aminosulfonyl)benzoic acid (4) (2 mmol) and N,N,N,N-tetramethyl-O-(1H-benzotriazol-1-yl)uranium hexafluorophosphate (HBTU) (2 mmol) in DMF(2 ml) was stirred at room temperature for 1 h. Then, a solution of the appropriate amine derivative (2 mmol) in TEA (2 mmol) was added dropwise. The reaction mixture was left overnight, then quenched with water (10 ml) and extracted with EtOAc (3 × 10 ml). The organic phase was dried with Na_2_SO_4_ and the solvent was removed *in vacuo*. The residue was purified by flash chromatography (DCM/MeOH 96:4), crystallized by treatment with a mixture of Et_2_O and EtOH (1:1) to give the desired final compounds **5b**, **5c**, **6a**, **7a–f,** and **8a–d** as white powders; the chemical characterisation of all re-synthesised compounds was in good agreement with literature (see Supporting Material).

### 2.2. Preparation of the bacterial CAs

The three bacterial enzymes were prepared accordingly to the procedure reported by our groups^24^. Briefly, the GeneArt Company (Invitrogen), specialised in gene synthesis, designed the genes encoding for the bacterial *α*, *β*, and γ- CAs. The BL21 DE3 competent cells (Agilent) were transformed with the expression vector pET15-b containing the gene encoding for one of the three CA-classes. Subsequently, bacterial cells were induced with 1 mM IPTG and, after 30 min, treated with 0.1 M ZnCl_2_. After 4 h, cells were harvested and disrupted by sonication at 4 °C. After centrifugation at 12,000×*g* for 45 min, the supernatant was incubated with His Select HF nickel affinity gel resin (Sigma) equilibrated in lysis buffer for 30 min. The protein was eluted with the wash buffer containing 200 mM imidazole. Collected fractions were dialysed against 50 mM Tris/HCl, pH 8. At this stage of purification, the protein was at least 95% pure.

### 2.3. Carbonic anhydrase inhibition assay

An Applied Photophysics stopped-flow instrument (Leatherhead, Surrey (UK)) has been used for assaying the CA catalysed CO_2_ hydration activity^25^. Phenol red (at a concentration of 0.2 mM) has been used as an indicator, working at the absorbance maximum of 557 nm, with 20 mM TRIS (pH 8.3) as buffer, and 20 mM NaClO_4_ (for maintaining constant the ionic strength), following the initial rates of the CA-catalysed CO_2_ hydration reaction for a period of 10–100 s. The CO_2_ concentrations ranged from 1.7 to 17 mM for the determination of the kinetic parameters (by Lineweaver–Burk plots) and inhibition constants. For each inhibitor, at least six traces of the initial 5–10% of the reaction have been used for determining the initial velocity. The un-catalysed rates were determined in the same manner and subtracted from the total observed rates. Stock solutions of inhibitor (10–100 mM) were prepared in distilled-deionized water, and dilutions up to 0.01 mM were done after that with the assay buffer. Inhibitor and enzyme solutions were preincubated together for 15 min at room temperature before assay, to allow for the formation of the E–I complex or the eventual active site-mediated hydrolysis of the inhibitor. The inhibition constants were obtained by non-linear least-squares methods using PRISM 3 and the Cheng-Prusoff equation, as reported earlier^26–28^, and represent the mean from at least three different determinations. The human CA isoforms were recombinant ones obtained in-house. All salts/small molecules were of the highest purity available, from Sigma-Aldrich (Milan, Italy).

### 2.4. Modelled structure of the opening of the active site of β–carbonic anhydrase from V. cholerae

The structure used to perform docking studies was built on different steps. First of all, the crystal structure of closed β-CA from *V. cholerae* was retrieved from the RCSB Protein Data Bank (PDB code 5CXK)^29^. The water molecules were discarded, then hydrogen atoms were added to the protein by using Discovery Studio 2.5.5 software^30^. In the second step, a superimposition of residues Cys42, Asp44, Arg46, His98, Cys101, Zn301 of β-CA from *V. cholerae* on corresponding residues Cys47, Asp49, Arg51, His103, Cys106, Zn228 of chain A of X-ray complex of AAZ with β-CA from the unicellular green alga *Coccomyxa* (*Co*-CA, PDB code 3UCJ)^31^ was performed using PyMOL software (https://pymol.org).

The above-mentioned superimposition was useful to modify the orientation of the side-chains of the two amino acids Asp44 and Arg46 of β-CA from *V. cholerae* using as template the conformation of Asp49 and Arg51 of β-CA from *Coccomyxa*. The modified structure of β-CA from *V. cholerae* was minimised using the conjugate gradient algorithm of NAMD. Minimisation was performed for 500 steps keeping all atoms fixed except for the backbones of residue 43–47 and the side-chains of Asp44 and Arg46: in detail, to maintain the correct orientation of carboxylate moiety of Asp44 and guanidinium group of Arg46 also these groups were kept fixed.

The atom types were assigned using force field CHARMM v22 and the atomic charges according to Gasteiger–Marsili method by Vega^32^. Furthermore, in the obtained structure, assuming a similar binding mode of the AAZ in both of the β-CAs, we inserted in the catalytic site of β-CA from *V. cholerae* the X-ray conformation of the acetazolamide retrieved from β-CA from *Coccomyxa*. The side-chains of the residues around the inhibitor AAZ have been minimised by LigandScout^33^.

### 2.5. Docking studies

The complex obtained was used to perform docking studies by GOLD software^34^ and the AAZ was discarded. The ligand structures were constructed by Vega 3.1.1 and the energy was minimised by using the Conjugate Gradient method (1000 steps). The minimised ligands were docked in their corresponding proteins using Gold Suite 5.0.1. The region of interest, which was used by the Gold programme, was defined as containing the residues within 10 Å of the original position of the acetazolamide in the model structure.

A scaffold constraint (penalty = 5.0) was used to restrict the solutions in which the sulfonamide moiety was able to coordinate the metal within the catalytic binding site. ChemPLP was chosen as the fitness function. The standard default settings were used in all the calculations and the ligands were submitted to 100 genetic algorithm runs. The “allow early termination” command was deactivated. Results differing by less than 0.75 Å in the ligand − all atom RMSD were clustered together. The conformations with the highest fitness values were chosen for both analysis and representation.

All obtained complexes were minimised keeping the Zn ion fixed and using the conjugated gradients algorithm by NAMD^35^. The results were displayed using the PyMOL software (https://pymol.org).

## 3. Results and discussion

### 3.1. Chemistry

**Scheme 1.**
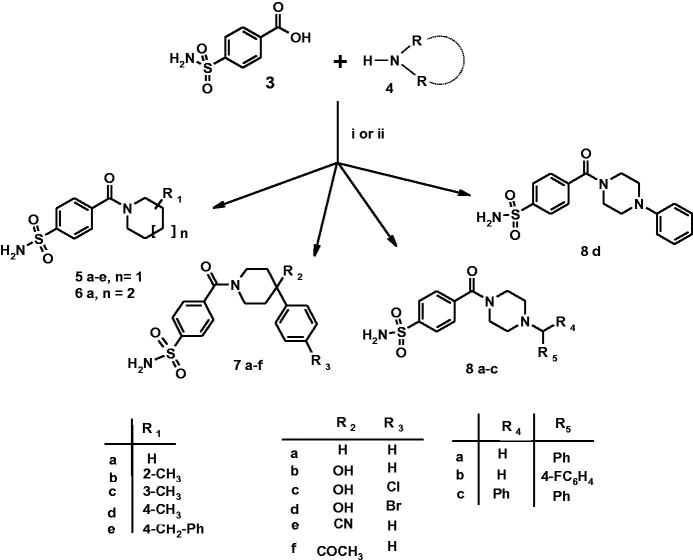
Reagents and conditions: (i) carbonyldiimidazole (DCI), THF, r.t., 3 h, then RR’NH, DMF r.t., 2 h; (ii) RR’NH, HBTU, DMF, TEA, r.t., overnight.

As displayed in Scheme 1, the studied 4-(cycloalkyl)-1-carbonylbenzenesulfonamides **5a–e**, **6a, 7a–f, 8a–d** have been re-synthesised following a synthetic procedure previously reported by us^23^; experimental details can be found in “Materials and Methods” section as well as in Supporting Material.

### 3.2. Carbonic anhydrase inhibition

The inhibitory effects of this series of sixteen 4-(cycloalkyl-1-carbonyl)benzenesulfonamide derivatives **5a–e**, **6a**, **7a–f**, **8a–c,** and **8d** were evaluated against VchCAα, VchCAβ, and VchCAγ by using the stopped-flow carbon dioxide hydrase assay. The obtained results are summarised in Table 1 and compared with the well-known inhibitor AAZ (**1**). Moreover, the *K*_i_ values measured against bacterial CAs were compared with an affinity towards selected human CA isoforms hCA I and hCA II^23^.

**Table 1.** Inhibition of hCA I, hCA II, VchCAα, VchCAβ, and VchCAγ for compounds **5a–e**, **6a**, **7a–f**, **8a–c** and **8d** and acetazolamide (**1**, AAZ) by a stopped flow CO_2_ hydrase assay.

*K*_i_ (nM)^a^					
Cmp	hCA I^b^	hCA II^c^	VchCAα	VchCAβ	VchCAγ
**5a**	9.2	5.7	843.2	>10,000	>10,000
**5b**	4.4	0.63	296.4	>10,000	>10,000
**5c**	187	6.1	528.8	>10,000	>10,000
**5d**	7.7	0.79	588.9	>10,000	>10,000
**5e**	75.5	1.5	241.9	3754	>10,000
**6a**	85.0	3.8	275.4	>10,000	>10,000
**7a**	1.7	0.5	43.3	3308	>10,000
**7b**	9.3	0.6	193.8	3714	>10,000
**7c**	6.6	0.6	45.1	3945	>10,000
**7d**	6.3	0.7	58.8	2776	>10,000
**7e**	6.5	0.6	89.9	806.4	>10,000
**7f**	7.7	0.6	72.1	6612	>10,000
**8a**	6.8	3.0	470.4	>10,000	>10,000
**8b**	0.69	0.5	95.7	5008	>10,000
**8c**	50.1	5.7	275.5	4816	>10,000
**8d**	91.1	2.0	47.0	4156	>10,000
**AAZ (1)**	250	12.1	6.8	451	473

^a^Mean from three different assays (errors were in the range of ±5–10% of the reported values). ^b,c^Data reported in reference^23^.

As reported in Table 1, all studied compounds proved to be inhibitors of the VchCAα and displayed *K*_i_s falling in a wide range of 43.3 and 843.2 nM. In terms of structure-affinity relationship considerations, the data collected in Table 1 suggested that the unsubstituted 4-(piperidine-1-carbonyl)benzenesulfonamide (**5a**), the three methyl-substituted analogues **5b**, **5c**, **5d** as well as cyclohomologue compound **6a** were less active than corresponding 4-aryl-substituted compounds **7a, 7 b, 7c, 7d, 7e,** and **7f**. Compound **7a** (*K*_i_ value of 43.3 nM) demonstrated the best activity having the phenyl ring linked to 4-position of piperidine nucleus. The introduction of an additional hydroxyl group, nitrile or acetyl functionalities at C–4 position of piperidine nucleus induces a weak decrease of affinity. The presence of 4′-chlorophenyl ring and hydroxyl group at 4-position of piperidine nucleus of compound **7c** afforded equi-active inhibitor of unsubstituted 4-phenyl analog **7a**. Among the series of benzenesulfonamides **8a–d** containing the piperazine core, the 4-(4-phenylpiperazine-1-carbonyl)benzenesulfonamide (**8d**) displayed the best inhibitory effect (*K*_i_ value of 47.0 nM), thus confirming that the presence of a bulky aromatic group anchored to the 4-position of cycloalkylamine nucleus generally improves the affinity against VchCAα. It is interesting to note the impact of the introduction of a methylene spacer between piperazine core and phenyl moiety: it was found a ten-fold reduction of activity of the compound 4-(4-benzylpiperazine-1-carbonyl)benzenesulfonamide (**8a**) (*K*_i_ = 470 nM) when compared to parent compound **8d**. This evidence might be due to a different binding within the catalytic cavity. Moreover, the introduction of a fluorine atom in the para position of the phenyl ring of benzylpiperazine-sulfonamide **8b** (*K*_i_ value of 95.7 nM) improves the affinity toward VchCAα. On the contrary, the presence of a benzhydryl moiety (compound **8c**, *K*_i_ = 275.5 nM) seems well tolerated to respect the benzyl-fragment (compound **8a**).

Concerning the activity on VchCAβ, several compounds proved to be inhibitors at low micromolar concentration showing inhibition data ranging from 806.4 nM to 6612 nM. Overall, the presence of a 4-aryl moiety resulted advantageous for inhibitory effects toward VchCAβ. The best affinity was found for 4-(4-cyano-4-phenyl-piperidine-1-carbonyl)benzenesulfonamide compound **7e** (*K*_i_ = 806.4 nM). The presence of other small substituents, such as acetyl for compound **7f** or hydroxyl group for compounds **7 b**, **7c**, and **7d** did not significantly influence the inhibitory effects toward VchCAβ. Moreover, the replacement of piperidine nucleus with piperazine one resulted in divergent effects: (a) it was found a completely loss of inhibitory activity for unsubstituted compound **8a**, whereas compounds **8b**, **8c**, and **8d** shared similar potency as moderate inhibitors of VchCAβ class.

On the contrary, all studied compounds were totally ineffective inhibitors against VchCAγ enzyme. Despite these compounds demonstrated interesting selectivity against VchCAα to respect β- and γ-classes, they proved to be active inhibitors of ubiquitously expressed hCAI and hCA II at low nanomolar concentration, thus impairing their employment as therapeutics in humans.

### 3.3. Modeling

In order to gain more information about the binding interaction of the most active compounds into the enzymatic catalytic site, we carried out a theoretical structural study for understanding and revealing the binding mode of inhibitors toward bacterial CA classes. The crystal structure of apoenzyme VchCAβ has been recently reported by Ferraroni et al. (PDB code 5CXK)^29^, whereas, the determined structures of VchCAα and VchCAγ from *V. cholerae* are not currently available. The crystal structures of β-CA are reported for other species^29^^,^^36–39^; these structural data furnished information about the homology of their 3-D folds as well as the shape of catalytic site, that displays the zinc coordinated by two cysteines, one-histidine and one-aspartic amino acid residue (the so-called “closed active site”). Particularly, for VchCAβs the catalytic zinc ion is coordinated by Cys42, Asp44, His98, and Cys101. (See schematic representation in Supporting Material). The catalytic site of VchCAβs assumes the so-called “closed active site” conformation as an inactive state. The pH-tuned movement of the Asp 44 residue from metal ion converts the “closed active site” to the “open active site”. Since the available 3 D structure the β-CA from *V. cholerae* was solved only in the “closed active site” configuration^29^, the rational design of novel inhibitors through a classical structure-based approach is currently hampered. To overcome the lack of this crucial structural information, in the first step of our theoretical study, we performed a simulation for the opening of the active site of β-CA from *V. cholerae*. In more details, we superimposed the 3 D X-ray structure of “closed active site” of β-CA from *V. cholerae* (PDB code 5CXK)^29^ to “open active site” of available β-CA from *Coccomyxa* in complex with inhibitor AAZ (**1**) (PDB code 3UCJ)^31^. Based on the conformation of side chain of crucial residues Asp49 and Arg51 of β-CA from *Coccomyxa*, we successively modified the orientation of the corresponding side-chain of Asp44 and Arg46, that are considered responsible for proton shuttling and “closed state” of β-CA. By assuming that inhibitor **1** displays a similar binding mode in these two β-CAs, we extracted AAZ from β-CA of *Coccomyxa* and then docked this inhibitor in the built 3 D structure of β-CA from *V. cholerae*; in the last step, we minimised all system. As a result, we obtained the hypothetical “*open active site*” of β-CA from *V. cholerae* as the “first in silico model”, useful for mapping the inhibitor binding interactions; our idea was to translate these data for understanding the inhibitory effects of the above mentioned sixteen 4-(cycloalkyl-1-carbonyl)benzensulfonamide derivatives (**5a–e**, **6a**, **7a–f**, **8a–d**) toward VchCAβ.

**Figure 2.**
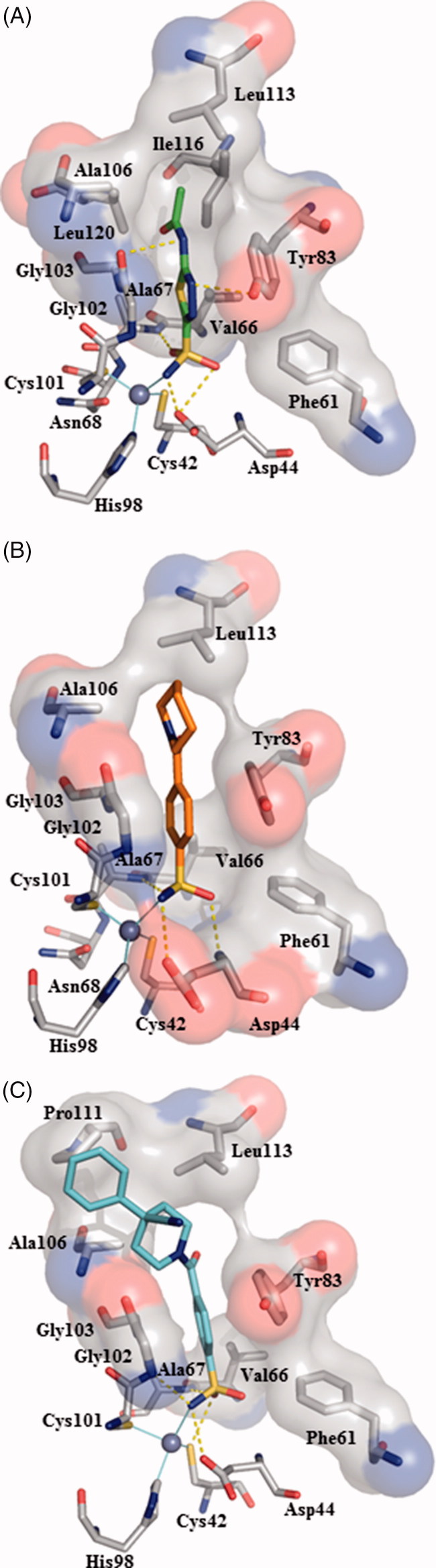
Docking poses into our “modelled” VchCAβ catalytic cavity for acetazolamide (A), sulfonamide derivatives **5a** (B) and **7e** (C). The interactions between the VchCAβ and inhibitors AAZ, **5a** and **7e** were examined using PyMOL and LIGPLUS^40^ software; residues involved in hydrophobic interactions and hydrogen bonds (yellow dashed lines) and Zn ion coordination are shown.

Figure 2 (A) displays AAZ (**1**) bound directly to the “hypothetic open” active site of β-CA from *V. cholerae*. As expected, the sulfonamide moiety anchors the catalytic zinc ion; additionally, the nitrogen of the N-acetamido group forms a hydrogen bond to the oxygen atom of Gly102, the thiadiazole ring establishes π/π interaction with Tyr83 which is reinforced by further hydrogen bond contacts between heterocyclic nitrogen atoms and hydroxyphenyl substituent. This network of interactions might explain the affinity for VchCAβ at submicromolar concentration (*K*_i_ value of 451 nM, Table 1), thus supporting the reliability of our modelled VchCAβ catalytic cavity. Then we studied the docking poses into our “modelled” VchCAβ catalytic cavity for all 4-(cycloalkyl-1-carbonyl)benzensulfonamide derivatives (**5a–e**, **6a**, **7a–f**, **8a–d**) bearing the sulfonamide as a minimal structural requirement to anchor the zinc ion in the deep catalytic site of all CAs.

In Figure 2(B), we described the best pose of prototype unsubstituted compound **5a** showing the ability to create a network of interactions with the deep area of the catalytic site near to zinc ion. As expected, the sulfonamide group is engaged in polar contacts with the CA cavity, whereas the remaining portion of molecule occupies the middle area establishing few hydrophobic interactions with residues Tyr83, Leu113, and Ala106. Therefore, we hypothesised that these interactions could be not sufficient to inhibit VchCAβ ( *K*_i_ >10,000 nM ). This consideration might be applicable to justify the low efficacy demonstrated by sulfonamide derivatives **5 b–d** and **6a** structurally related to parent compound **5a**. Interestingly, the best active inhibitor **7e** (*K*_i_ = 806.4 nM, see Figure 2(C)) demonstrated the ability to form additional interactions with a cluster of hydrophobic residues Ala106, Pro111, and Leu 113 when compared with unsubstituted analog **5a**, thus suggesting that the 4-phenyl substituent of compound **7e** is a crucial fragment to make more favourable hydrophobic contacts in the top of the cavity. This result indicates that the ligands having a good affinity towards VchCAβ are expected to establish additional hydrogen bonding interactions or hydrophobic contacts in the middle or top region of the enzymatic cavity.

## 4. Conclusions

In conclusions, a small series of benzenesulfonamides has been screened as inhibitors of *V. cholerae* carbonic anhydrases. Compound **7e** demonstrated interesting affinity against VchCAα and VchCAβ with *K*_i_ values of 89.9 and 806.4 nM, respectively. The predicted binding mode of compound **7e** in the modelled catalytic site of VchCAβ suggests that the introduction of an extra aromatic ring might improve the contacts in the top area of the catalytic cavity, thus furnishing suggestions for the rational design of new compounds targeting *V. cholerae* CAs.

## Supplementary Material

Supplemental Material
