# Supplementary material for: Seeking new approach for therapeutic treatment of cholera disease via inhibition of bacterial carbonic anhydrases: experimental and theoretical studies for sixteen benzenesulfonamide derivatives
Source: J Enzyme Inhib Med Chem. 2019 Jul 8;34(1):1186–92. doi: 10.1080/14756366.2019.1618292 (PMC6691843; doi:10.1080/14756366.2019.1618292)
Supplement: Supplemental Material [file IENZ_A_1618292_SM6515.docx]

Supporting Material

Seeking new approach for therapeutic treatment of cholera disease via inhibition of bacterial carbonic anhydrases: experimental and theoretical studies for sixteen benzenesulfonamide derivatives

Rosaria Gitto, Laura De Luca, Francesca Mancuso, Sonia del Prete, Daniela Vullo, Claudiu T. Supuran, Clemente Capasso

**Contents**

1. Figure 1. VchCAβ active site (PDB code 5CXK)
2. Chemical Characterization of 4-(cycloalkyl-1-carbonyl)benzenesulfonamide derivatives **5a-e, 6a, 7a-f, 8a-c** and **8d**


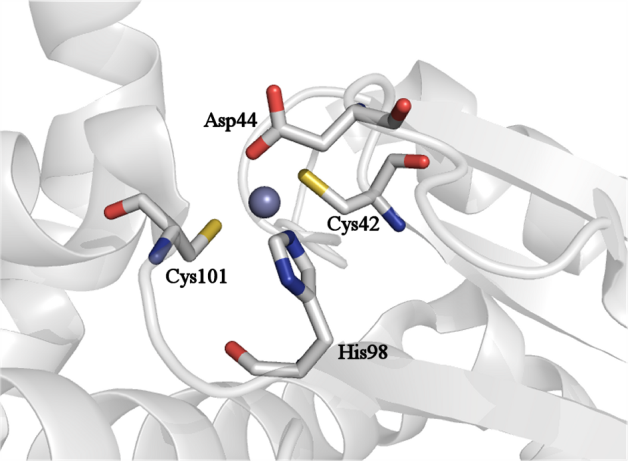


1. **Figure 1. Structural representation of active site of VchCAβ (PDB code 5CXK)**[**^32^**](#_ENREF_32) **exhibiting a ‘closed’ conformation**
2. **Chemical Characterization of 4-(cycloalkyl-1-carbonyl)benzenesulfonamide derivatives 5a-e, 6a, 7a-f, 8a-c and 8d**

***4-(Piperidine-1-carbonyl)benzenesulfonamide (5a)***

Yield: 40%; M.p.: 206-207°C; R_f_ = 0.45. ^1^H-NMR (DMSO-d_6_): (δ) 1.43-3.57 (m, 10H), 7.45 (bs, 2H, NH_2_), 7.53 (d, J=8.2, 2H, ArH). 7.83 (d, J=8.2, 2H, ArH). Anal. Calcd for C_12_H_16_N_2_O_3_S: C: 53.71; H:6.01; N:10.44. Found: C:53.81; H: 6.11; N 10.54.

***4-(2-Methylpiperidine-1-carbonyl)benzenesulfonamide (5b)***

Yield: 60%; M.p.: 186-187°C; R_f_ = 0.47. ^1^H-NMR (DMSO-d_6_): (δ) 1.15 (d, J=7.1, 3H, CH_3_), 1.43-3.40 (m, 9H), 7.42 (bs, 2H, NH_2_), 7.50 (d, J=8.2, 2H, ArH). 7.83 (d, J=8.2, 2H, ArH). Anal. Calc for C_13_H_18_N_2_O_3_S: C:55.30; H: 6.43; N:9.92. Found: C:55.35; H:6.40; N:9.97.

***4-(3-Methylpiperidine-1-carbonyl)benzenesulfonamide (5c)***

Yield: 35%; M.p.: 190-191°C; R_f_ = 0.49. ^1^H-NMR (DMSO-d_6_): (δ) 1.06 (d, J=7.1, 3H, CH_3_), 1.10-2.94 (m, 9H), 7.43 (bs, 2H, NH_2_), 7.52 (d, J=8.2, 2H, ArH), 7.84 (d, J=8.2, 2H, ArH). Anal. Calcd for C_13_H_18_N_2_O_3_S: C:55.30; H: 6.43; N:9.92. Found: C:55.40; H:6.53; N:10.02.

***4-(4-Methylpiperidine-1-carbonyl)benzenesulfonamide (5d)***

Yield: 40%; M.p.: 212-213°C; R_f_ = 0.51. ^1^H-NMR (DMSO-d_6_): (δ) 0.90 (d, J=7.0, 3H, CH_3_), 1.06-4.44 (m, 9H), 7.44 (bs, 2H, NH_2_), 7.53 (d, J=8.2, 2H, ArH). 7.84 (d, J=8.2, 2H, ArH). Anal. Calcd for C_13_H_18_N_2_O_3_S: C:55.30; H:6.43; N:9.92. Found: C:55.40; H:6.40; N:9.80.

***4-(4-Benzylpiperidine-1-carbonyl)benzenesulfonamide (5e)***

Yield 44%; M.p.: 156-157°C; R_f_ = 0.59. ^1^H-NMR (DMSO-d_6_): (δ) 1.12-1.66 (m, 4H), 2.52 (m, 2H, CH_2_Ph), 2.62-2.44 (m, 5H), 7.15-7.16 (m, 3H, ArH), 7.24-7.27 (m, 2H, ArH), 7.42 (bs, 2H, NH_2_), 7.52 (d, J=8.2, 2H, ArH), 7.84 (d, J=8.2, 2H, ArH). Anal. Calcd for C_19_H_22_N_2_O_3_S: C:63.66; H:6.19; N:7.81. Found: C:63.56; H:6.09; N:7.71.

***4-(Azepane-1-carbonyl)benzenesulfonamide (6a)***

Yield: 30%; M.p.: 200-201 °C; R_f_ = 0.53. ^1^H-NMR (DMSO-d_6_): (δ) 1.50-3.53 (m, 12H), 7.43 (bs, 2H, NH_2_), 7.51 (d, J=7.6, 2H, ArH). 7.83 (d, J=7.6, 2H, ArH). Anal. Calcd for C_13_H_18_N_2_O_3_S: C:55.30; H:6.43; N:9.92. Found: C:55.40; H: 6.53, N:9.82.

***4-(4-Phenylpiperidine-1-carbonyl)benzenesulfonamide (7a)***

Yield: 73%; M.p.: 222-223°C; R_f_ = 0.55. ^1^H-NMR (DMSO-d_6_): (δ) 1.86-4.60 (m, 9H), 7.18-7.28 (m, 5H, ArH), 7.45 (s, 2H, NH_2_), 7.61 (d, J=8.00, 2H, ArH), 7.86 (d, J=8.00, 2H, ArH). Anal. Calcd for C_18_H_20_N_2_O_3_S: C:62.77; H:5.85; N:8.13. Found: C:63.10; H:5.65; N:8.47.

***4-(4-Hydroxy-4-phenylpiperidine-1-carbonyl)benzenesulfonamide (7b)***

Yield: 34%; M.p.: 135-137°C; R_f_ = 0.37. ^1^H-NMR (DMSO-d_6_): (δ) 1.51-4.45 (m, 8H), 5.20 (bs, 1H, OH), 7.18-7.32 (m, 3H, ArH), 7.45 (bs, 2H, NH_2_), 7.51 (m, 2H, ArH), 7.63 (d, J=8.2, 2H, ArH), 7.86 (d, J=8.2, 2H, ArH). Anal. Calcd for C_18_H_20_N_2_O_4_S: C:59.98; H:5.59; N:7.77. Found: C:60.18; H:5.79; N:7.87.

***4-[4-(4-Chlorophenyl)-4-hydroxypiperidine-1-carbonyl]benzenesulfonamide (7c)***

Yield: 31%; M.p.: 215-217°C; R_f_ = 0.38. ^1^H-NMR (DMSO-d_6_): (δ) 1.52-4.44 (m, 8H), 5.31 (bs, 1H, OH), 7.36 (d, J=8.5, 2H, ArH), 7.44 (bs, 2H, NH_2_), 7.54 (d, J=8.5, 2H, ArH), 7.63 (d, J=7.6, 2H, ArH), 7.86 (d, J=7.6, 2H, ArH). Anal. Calcd For C_18_H_19_ClN_2_O_4_S: C:54.75; H:4.85; N:7.09. Found: C:54.93; H:5,13; N: 7.37.

***4-[4-(4-Bromophenyl)-4-hydroxy-piperidine-1-carbonyl]benzenesulfonamide (7d)***

Yield: 35%; M.p.: 219-220°C; R_f_ = 0.40. ^1^H-NMR (DMSO-d_6_): (δ) 1.48-4.45 (m, 8H), 5.31 (s, 1H, OH), 7.44 (bs, 2H, NH_2_), 7.49-7.52 (m, 4H, ArH), 7.65 (d, J=8.8, 2H, ArH), 7.87 (d, J=8.8, 2H, ArH). Anal. Calcd for C_18_H_19_BrN_2_O_4_S: C:49.21; H:4.36; N:6.38. Found: C:49.58; H:4.67; N:6.44.

***4-(4-Cyano-4-phenylpiperidine-1-carbonyl)benzenesulfonamide (7e)***

Yield: 22%; M.p.: 285-286°C; R_f_ = 0.59. ^1^H-NMR (DMSO-d_6_): (δ) 2.07-4.67 (m, 8H), 7.35-7.42 (m, 3H, ArH), 7.42 (bs, 2H, NH_2_), 7.45-7.47 (m, 4H, ArH), 7.66 (d, J=8.2, 2H, ArH), 7.87 (d, J=8.2, 2H, ArH). Anal. Calcd for C_19_H_19_N_3_O_3_S: C: 61.77; H:5.18; N:11.37. Found: C:6.83; H:5.47; N:11.62.

***4-(4-Acetyl-4-phenyl-piperidine-1-carbonyl)benzenesulfonamide (7f)***

Yield: 20%; M.p.: 233-234°C; R_f_ = 0.52. ^1^H-NMR (DMSO-d_6_): (δ) 1.89 (s, 3H, CH_3_), 1.96-3.92 (m, 8H), 7.28-7.38 (m, 5H, ArH), 7.44 (bs, 2H, NH_2_), 7.55 (d, J=8.2, 2H, ArH), 7.83 (d, J=8.2, 2H, ArH). Anal. Calcd for C_20_H_22_N_2_O_4_S: C:62.16; H:5.74; N:7.25. Found: C:62.10; H:5.84; N:7.15.

***4-(4-Benzylpiperazine-1-carbonyl)benzenesulfonamide (8a)***

Yield 40%; M.p.: 204-205°C; R_f_ = 0.43. ^1^H-NMR (DMSO-d_6_): (δ) 2.32- 3.60 (m, 8H), 3.47 (s, 2H, CH_2_Ph), 7.22-7.26 (m, 5H, ArH), 7.44 (bs, 2H, NH_2_), 7.56 (d, J=8.2, 2H, ArH), 7.83 (d, J=8.2, 2H, ArH). Anal. Calcd for C_18_H_21_N_3_O_3_S: C:60.15; H:5.89; N:11.69. Found: C:60.25; H:5.99; N:11.99.

***4-[4-[(4-Fluorophenyl)methyl]piperazine-1-carbonyl]benzenesulfonamide (8b)***

Yield 30%; M.p. 206-207°C; R_f_ = 0.45. ^1^H-NMR (CDCl_3_): (δ) 2.35-3.62 (m, 8H), 2.49 (s, 2H, CH_2_Ph), 7.11-7.36 (m, 4H, ArH), 7.46 (bs, 2H, NH_2_), 7.57 (d, J=8.2, 2H, ArH), 7.85 (d, J=8.2, 2H, ArH). Anal. Calcd for C_18_H_20_FN_3_O_3_S: C:57.28; H:5.34; N:11.13. Found: C:57.55; H:5.43; N:11.34.

***4-(4-Benzhydrylpiperazine-1-carbonyl)benzenesulfonamide (8c)***

Yield 60%; M.p.: 228-230°C; R_f_ = 0.58. ^1^H-NMR (DMSO-d_6_): (δ) 2.26-4.32 (m, 9H), 7.16-7.39 (m, 10H, ArH), 7.41(bs, 2H, NH_2_), 7.52 (d, J=8.2, 2H, ArH), 7.81 (d, J=8.2, 2H, ArH). ^13^C-NMR (DMSO-d_6_): (δ) 172.8, 149.9, 147.6, 144.14, 133.8, 132.8, 132.19, 131.08, 130.8, 79.9, 79.8. Anal. Calcd for C_24_H_25_N_3_O_3_S: C:66.18; H:5.79; N:9.65. Found: C:66.28; H:5.69; N:9.55.

***4-(4-Phenylpiperazine-1-carbonyl)benzenesulfonamide (8d)***

Yield 84%; M.p.: 231-232°C; R_f_ = 0.59. ^1^H-NMR (DMSO-d_6_): (δ) 3.10-3.89 (m, 8H), 6.80-7.24 (m, 5H, ArH), 7.42 (bs, 2H, NH_2_), 7.58 (d, J=8.2, 2H, ArH), 7.84 (d, J=8.2, 2H, ArH). Anal. Calcd for C_17_H_19_N_3_O_3_S: C:59.11; H:5.54; N:12.16. Found: C:58.81; H:5.24; N:12.06.
